# Visualization of estimated prevalence of CES-D positivity accounting for background factors and AIS scores

**DOI:** 10.1038/s41598-022-22266-1

**Published:** 2022-10-21

**Authors:** Takashi Matsuyama, Akira Narita, Masaki Takanashi, Mana Kogure, Shuichi Sato, Tomohiro Nakamura, Hideo Nakane, Soichi Ogishima, Fuji Nagami, Naoki Nakaya, Kozo Tanno, Takao Imaeda, Atsushi Hozawa

**Affiliations:** 1 Toyota Central Research and Development Laboratories Inc., Nagakute, 480-1192 Japan; 2 Tohoku Medical Megabank Organization, Tohoku University, Sendai, 980-8573 Japan; 3 Iwate Tohoku Medical Megabank Organization, Iwate Medical University, Yahaba-Cho, Iwate, 028-3694 Japan

**Keywords:** Epidemiology, Population screening

## Abstract

Development of methods for population screening is necessary to improve the efficiency of secondary prevention of diseases. Until now, a common cutoff has been used for all people in the data set. However, if big data for health information can be used to modify individual cutoffs according to background factors, it may avoid wasting medical resources. Here we show that the estimated prevalence of the Center for Epidemiologic Studies Depression Scale positivity can be visualized by a heatmap using background factors from epidemiological big data and scores from the Athens Insomnia Scale. We also show that cutoffs based on the estimated prevalence can be used to decrease the number of people screened without decreasing the number of prevalent cases detected. Since this method can be applied to the screening of different outcomes, we believe our work can contribute to the development of efficient screening methods for various diseases.

## Introduction

Secondary prevention of depression may be implemented in the general population since depression is deemed treatable. In secondary prevention of depression, people are usually screened using a self-assessment scale; thereafter, those who get a certain risk score are further diagnosed by a physician. The Center for Epidemiologic Studies Depression (CES-D)^1^ scale has been developed as a screening tool for depression in the general population. A previous systematic review^2^ reported that CES-D has a pooled sensitivity of 0.87 and a pooled specificity of 0.70 at a cutoff value of 16 points^3^ in terms of screening the general population. This scale possesses the same or better performance as compared to other self-assessment scales^2^.

Depression screening has been widely used while its guidelines differ from country to country. In the United States, screening for the general population is recommended in the primary care setting and in a well-followed environment^4^, while it is not recommended in Canada due to concerns of low cost-efficiency^5^. In the United Kingdom, screening is only recommended to high-risk people^6^. In Japan, annual screening of employees for depression in the workplace using self-assessment scales has become mandatory since 2015^7^.

The global prevalence of depression in the general population was estimated to be 4.4% in 2015^8^. Since many false positives are subjected to the detailed examination in screening for population who have a low probability of being CES-D positive resulting in waste of medical resources, two approaches addressing this problem have been identified. One approach is reducing the screening sensitivity and increasing its specificity, thus limiting the number of people who underwent the detailed examinations. The second approach is stratifying people by background factors and selecting a screening cutoff level for subgroups with different backgrounds^9,10^. Here we examined this possibility by visualizing the estimated prevalence of CES-D positivity with a combination of the Athens Insomnia Scale^11^ (AIS) and background factors when screening with CES-D positivity as an outcome.

The goal of this study is to propose a method to explicitly use epidemiological big data for efficient disease screening. Currently, artificial intelligence (AI) diagnostics based on deep learning from multimodal big data is being developed for various diseases, especially cancer^12^, but the process of diagnostics remains a “black box” due to the complex algorithms involved^13^. For example, a method to determine skin cancer from dermatoscope images by Deep Neural Network (DNN) has been proposed^14^, but it is difficult to prove its validity because various parameters such as the number of intermediate layers and nodes to achieve high determination accuracy must be determined by trial and error. Briefly, in deep learning-based AI diagnostics such as DNN, the logic of learning is not very complex, but the prediction rules represented in the model after learning have a level of complexity that is not understandable to humans. Meanwhile, in nasopharyngeal carcinoma, the improvement in screening efficiency was achieved by stratifying the population with high positive predictive value (PPV) by biomarkers and genetic risk factors^15^. In depression, which still has no clear physical basis and lacks biomarkers^16^, the use of epidemiological big data is appropriate, and visualizing the big data information is indispensable. In the proposed method, the impact of background factors in the epidemiological data may be compressed using a simple linear regression model with stratification by epidemiological risk factors and AIS scores as the only explanatory variables by visualizing a series of prevalence estimates of CES-D as a spectrum and setting appropriate screening cutoffs. In contrast to AI using deep learning, this method clearly explains the cutoff selection criteria.

## Results

**Figure 1:**
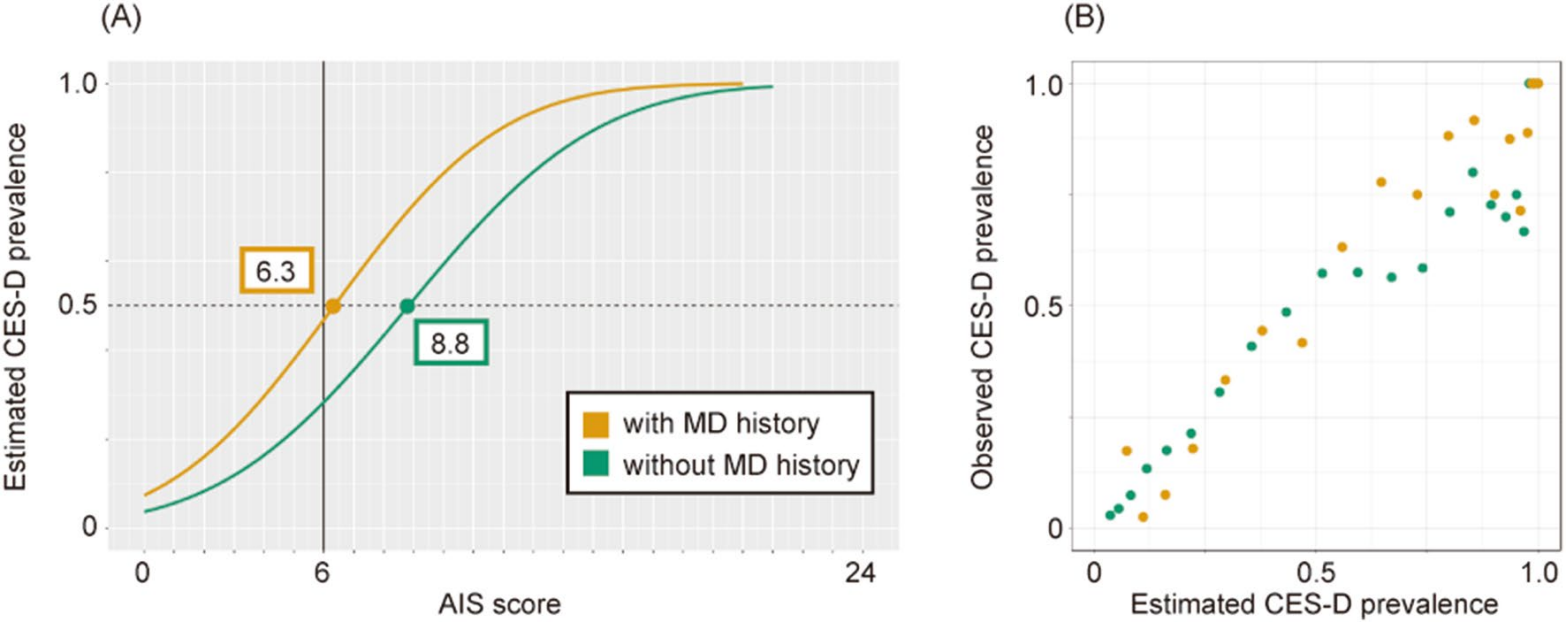
(**A**) Probit regression model of estimated prevalence of Center for Epidemiologic Studies Depression (CES-D) in subgroups stratified by history of mental disorder. The subgroup without MD, P (CES-D positive | AIS = x, mental disorder = 0), is shown in green, while that with MD, P (CES-D positive | AIS = x, mental disorder = 1), is in orange. An estimated prevalence of 50% is shown by the horizontal dotted line, as well as AIS score with a 50% of CES-D prevalence (ACP50), which is the intersection of this line with the graphs representing each model. (**B**) Scatter plot of the estimated and observed prevalence of CES-D positivity at AIS score. Each point indicates the respective AIS score (Supplementary Table 3). The correlation coefficient was R = 0.962, *p* < 0.01 for the subgroup with MD, and R = 0.962, *p* < 0.01 without MD.

The participants were stratified by mental disorder (MD) history to construct a model in each stratum (Fig. 1A). The prevalence of CES-D positivity estimated from the models increased as the AIS score increased, reflecting the observed prevalence (Fig. 1B).

The AIS score at which the prevalence of CES-D positivity be estimated 50% was defined as ACP50 and used as the cutoff. The ACP50 in the group without MD was 8.8 points, whereas that with MD was 6.3 points (Fig. 1 and Supplementary Table 1). Using ACP50 as the cutoff value for sleep disorders to divide these groups into subgroups of "above" if the AIS score was equal to or above the cutoff value and "below" if it was not, the prevalence of each subgroup was calculated (Table 1).

**Table 1:** Prevalence of Center for Epidemiologic Studies Depression positivity in each subgroup stratified by history of mental disorder and sleep disorders.

Stratification			Number of people		Prevalence
Mental disorder	Sleep disorder		Total	CES-D positives	
	Cutoff	Subgroup			
–			8440	1593	0.189
Without MD	–	Total	8024	1409	0.176
	ACP50 (8.8)	Below	7504	1091	0.145
		Above	520	318	0.612
MD	–	Total	416	184	0.442
	ACP50 (6.3)	Below	256	60	0.234
		Above	160	124	0.775

The prevalence of CES-D positivity in the crude population was 18.9%. Meanwhile, with MD stratification, the prevalence of the subgroup without MD was 17.6%, while that with MD was 44.2% (Table 1). In the subgroup without MD divided by ACP50, the prevalence of subgroups below ACP50 was 14.5%, whereas that above ACP50 was 61.2% (Table 1). When the subgroup with MD is divided by ACP50, the prevalence of subgroups below ACP50 was 23.4%, while that of the subgroup above ACP50 was 77.5% (Table 1).

**Figure 2:**
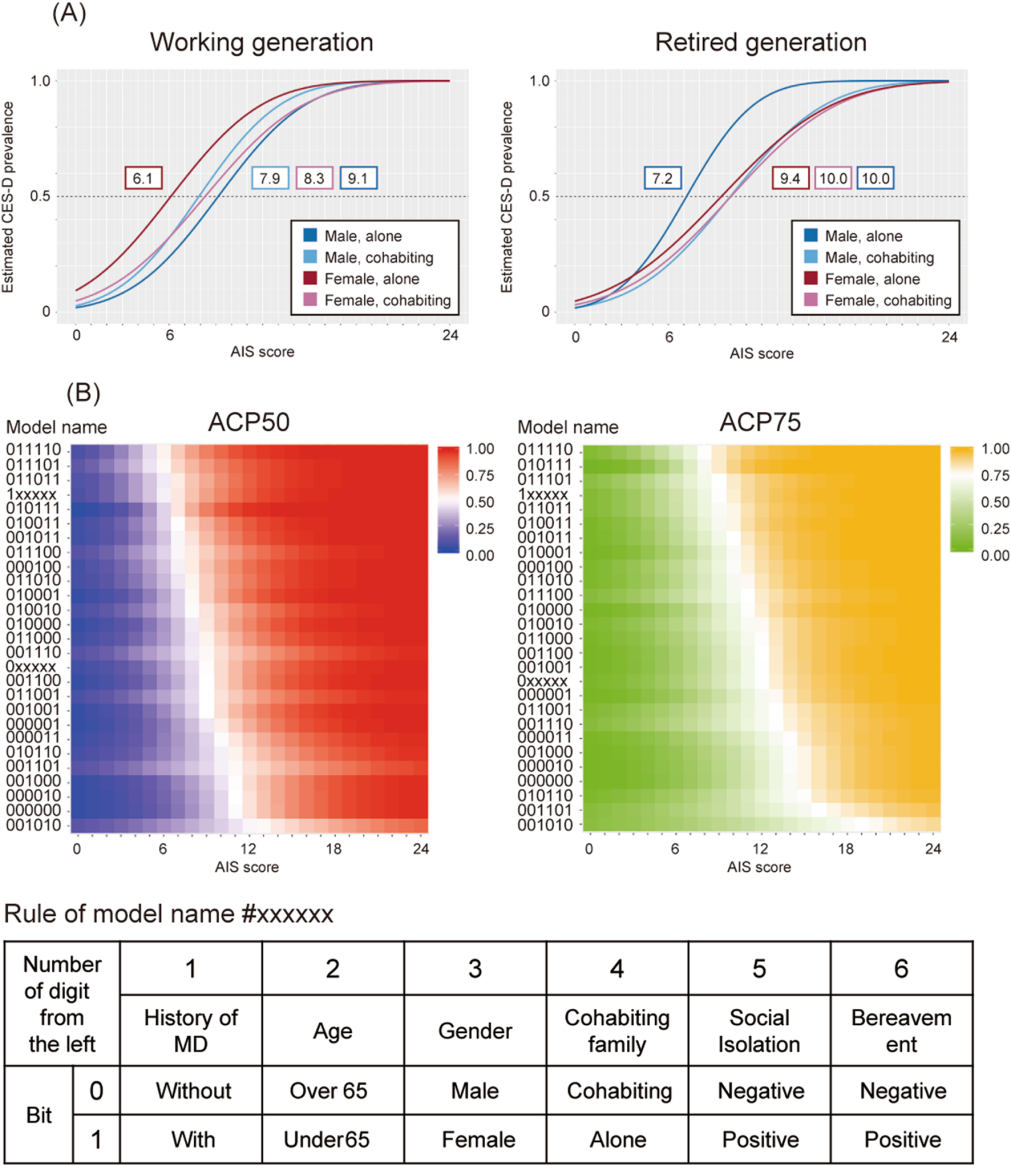
Probit regression models of estimated prevalence of Center for Epidemiologic Studies Depression (CES-D) score stratified by multiple risk factors for depression. (**A**) Graphs of estimated prevalence models P (CES-D positive | AIS = x, mental disorder = 0, stratified by age, sex, and cohabiting family). The graph representing the working generation is shown on the left, while the same for the retired generation is presented on the right. The ACP50 value of each subgroup is shown in the color frame. (**B**) Heatmaps of estimated prevalence models P (CES-D positive | AIS = x, mental disorder = 0, stratified by age, sex, cohabiting family, social isolation, and bereavement). The horizontal axis of the heatmaps in each panel indicates the AIS score, and names of each model are depicted along the left axis of each heat map. The spectra arranged in ascending order by ACP50 and ACP75 values are shown on the left and right graphs, respectively. For comparison, the spectra of the subgroups with and without MD are also presented in Fig. 1 (1xxxxx and 0xxxxx, respectively).

In the subgroup without MD, the estimated prevalence of CES-D positivity and the ACP50 of each subgroup stratified by multiple risk factors for depression were visualized via AIS score (Fig. 2). First, when we stratified the subgroup without MD into eight subgroups by sex, age, and cohabiting family members, each subgroup was modeled and visualized using a graph (Fig. 2A). Next, when we stratified the subgroup without MD into 32 subgroups by sex, age, cohabiting family, social isolation, and bereavement, each subgroup was modeled. Two models could not be constructed, and five models had a coefficient of *p* > 0.05 that did not satisfy statistical significance. These seven models were excluded from the analysis, two additional models of subgroups with and without MD were added, and the 27 models were visualized as a spectrum using a heatmap (Fig. 2B).

When ACP50 was used as a cutoff, the PPV of CES-D positivity was 63.7% when stratified by three risk factors, and 65.4% when stratified by five risk factors (Table 2). When ACP75 was used as a cutoff, the PPV was 76.5% when stratified by five risk factors (Table 2). The total number of people in the categories above ACP50 increased by 66 from 416 when stratified by the three risk factors to 482 when stratified by the five risk factors, while the number of CES-D-positive cases increased by 50 from 265 to 315. When ACP75 was used for the cutoff, the PPV was 76.5%, total number was 132, and number of CES-D positive cases was 101.

**Table 2:** Prevalence of Center for Epidemiologic Studies Depression positivity in each category classified by ACP50 or ACP75.

Stratification			Number of people		Prevalence
Risk factor	Sleep disorder		Total	CES-D Positives	
	Cutoff	Subgroup			
–	–	Total	7021	1222	0.174
Sex, Age, Cohabiting Family (Fig. 2A)	ACP50	Below	6605	957	0.145
		Above	416	265	0.637
Sex, Age, Cohabiting Family, Social Isolation, Bereavement (Fig. 2B)	ACP50	Below	6539	907	0.139
		Above	482	315	0.654
	ACP75	Below	6889	1121	0.163
		Above	132	101	0.765

The estimated prevalence in the subgroup without MD at an AIS score of 6, which is considered a category of sleep disorder, P (CES-D positive | AIS = 6, mental disorder = 0) was 0.283 (Table 3). Using ACP 28.3 as the cutoff, the PPV for this subgroup was 44.9% without stratification, 48.0% when stratified by the three risk factors, and 47.9% when stratified by the five risk factors (Table 3). In the group without MD, the total number of people in the categories above ACP28.3 was 1555 without stratification, and that was 1349 when stratified by the three risk factors and 1369 when stratified by the five risk factors. The number of people with CES-D prevalence in this category was 698 without stratification, and that was 648 when stratified by the three risk factors and 656 when stratified by the five risk factors (Table 3). On the other hand, when ACP26 was used as the cutoff in subgroups stratified by three factors, 662 CES-D-positive individuals were included in 1407 above ACP26, and when ACP25 was used as the cutoff in subgroups stratified by five factors, 696 CES-D-positive individuals were included in 1507 above ACP25 (Table 3).

**Table 3:** Prevalence of Center for Epidemiologic Studies Depression positivity in each category divided by sleep disorder cutoff.

Stratification			Number of people		Prevalence
Risk factor	Sleep disorder		Total	CES-D Positives	
	Cutoff	Subgroup			
–	AIS 6 points	Below	5466	524	0.096
		Equal or above	1555	698	0.449
Sex, age, and cohabiting family	ACP26	Below	5614	560	0.100
		Above	1407	662	0.471
	ACP28.3	Below	5672	575	0.101
		Above	1349	647	0.480
Sex, age, cohabiting family, social isolation, and bereavement	ACP25	Below	5514	526	0.095
		Above	1507	696	0.462
	ACP28.3	Below	5652	566	0.100
		Above	1369	656	0.479

## Discussion

First, the validity of this method was investigated. Positive predictive value is the probability that people with a positive screening test truly have a disease. Since the PPV varies with the prevalence of an inspected population, we examined the possibility of increasing PPV and improving screening efficiency by estimating the prevalence for each population with different background factors and setting a cutoff value based on this estimated prevalence. Using the binarized CES-D score according to a cutoff of 16 points, a surrogate outcome for the depression, as the objective variable and AIS score of the insomnia index as the explanatory variable, a developed model was constructed to estimate the prevalence of CES-D positivity for efficient population screening.

Insomnia is an operational diagnostic symptom of depression in Diagnostic and Statistical Manual of Mental Disorders, Fifth Edition (DSM-V)^17^. Several systematic reviews have strongly suggested that insomnia is a risk factor for depression^18,19^ and treatment guidelines have been established^20^. Commonly used scales of sleep disorders include the AIS, which was used in the WHO Worldwide Project on Sleep and Health^21^. AIS is measured in the range of 0 to 24 points, and the cutoff level for insomnia is usually 6 points^22^. The prevalence estimated in this model from the AIS score effectively reflected the observed prevalence of CES-D (Fig. 1B).

Our goal was to construct a model to determine what number of AIS scores would result in an arbitrary prevalence of CES-D positivity, for example 50% or 75% in this study. Therefore, we referred to the case of estimating the median effective dose, ED50, from the experimental values of the drug dose and the effect. The probit model is more accurate than the logit model when obtaining a dose–response curve based on the distribution for the dose of the drug that elicits the biological response^23^. In this study, the CES-D prevalence estimated from the probit model with the AIS score as the explanatory variable was consistent with the observed prevalence (Fig. 1B). The probit model estimates probabilities assuming that the error term in the estimating equation follows a normal distribution. Since the normal distribution is a continuous-type probability distribution that applies to many situations in nature and human society, the probit model was considered relatively more reasonable than the logit model^24^.

Background factors used for stratification, such as history of MD^6^, age^8^, sex^8^, cohabiting with family^25^, social isolation^26^, and bereavement, are factors strongly associated with depression. The factors of mental disorder history, working generation, being female, living alone, social isolation, and bereavement increase the MD risk. The risks identified in the previous studies were also considered in our dataset, whereas even with over 8,000 people in the dataset, we were not able to construct a model for the subgroup 011111 with the five high-risk categories except MD history (Supplementary Table 2). All people in the four subgroups with the lowest AIS scores (011110, 011101, 011011, and 010111) for ACP50 that showed a risk comparable to the MD subgroup were of the working age women and possessed three of the four other risks. The heatmap also demonstrates that the risk is higher for people with coinciding prior risk factors for CES-D including age.

Further, we investigated whether stratification could reduce the target population for screening. Among the 1,555 patients with the commonly used AIS of 6 points^22^ or higher, 698 were CES-D positive with a prevalence of 0.449. The estimated prevalence for CES-D at 6 AIS points was 0.283 (Table 3). The ACP required to detect 698 CES-D-positive individuals is ACP26 stratified by 3 levels, which detects 662 (94.8% of 698) CES-D-positive individuals from 1407 (90.4% of 1555), or ACP25 stratified by 5 levels, which detects 696 (99.7% of 698) CES-D-positive individuals from 1507 (96.9% of 1555). At ACP 28.3, when stratified by the three levels, 1349 individuals (86% of 1555) were needed to detect almost the same number [647 (93% of 698)] of CES-D positives. When stratified by five levels, 1369 individuals (88% of 1555) were needed to detect 656 (94% of 698) of them. These results indicate how to detect a similar number of positive people from a smaller number of screened people, thus making the screening more efficient.

As shown in Fig. 2, it is also possible to reduce the number of screened people by tuning the PPV with a cutoff value based on estimated prevalence. The PPV increased from 65.4% to 76.5% by changing the cutoff value from ACP50 to ACP75 when stratified by five risk factors, whereas the number of screened people decreased from 482 to 132, and the number of CES-D positive cases decreased from 315 to 101 (Table 2). These changes reflect the trade-off between the cost-efficiency of screening and the potential number of unaccounted patients.

## Conclusion

In this study, we showed that background information and AIS scores obtained from epidemiological big data can be compressed into prevalence of CES-D positivity. Furthermore, the number of people screened can be reduced using prevalence-based cutoffs while maintaining the same number of detected CES-D prevalent cases. Herein, with the high-risk factor hierarchy used for stratification and ACP setting, the subgroups with higher prevalence could be classified. Based on these results the potential for improving the efficiency of depression screening was demonstrated, where the screening cutoff can be adjusted according to available medical resources. Changing the cutoff of risk factors according to background factors and creating a heatmap for visualizing the results are the basic achievements of this study. Even for diseases for which biomarkers are not readily available, the versatility of epidemiological risk factors to visualize prevalence and determine cutoff values may increase PPV in a population. The prevalence of CES-D positivity as an outcome was used in our model instead of the prevalence of depressed patients; thus, the efficiency of depression screening may differ from the reality. Since the results of the analysis are based on data from a single cohort of residents in a particular region of Japan, it is necessary to verify the results in other cohorts and countries.

Studies have been conducted to improve the efficiency of secondary prevention by using different cutoffs for populations with different background factors, and these studies may be greatly advanced by incorporating approaches that utilize epidemiological big data. This method may be applied to the screening of other outcomes. We believe that it can contribute to the development of efficient screening methods based on the availability of big data. The validity of this method will be verified with various data sets of health big data being developed around the world, including genetic information.

## Methods

### Objects under study and stratification process

The Great East Japan Earthquake and the accompanying tsunami on March 11, 2011, have resulted in 15,897 deaths and 2534 missing persons^27^. Thus, Tohoku Medical Megabank community-based cohort study was launched in 2013 to investigate the long-term health effects of these disasters on the local population^28^. Approval for the study was obtained from the Institutional Review Board of Tohoku University. All participants gave written informed consent. This study was conducted according to the principles expressed in the Declaration of Helsinki. Among the 9815 participants in the Type I survey^29^ of this cohort used in the previous study^30^, 8440 participants who answered both the CES-D and AIS questionnaires were stratified by their self-reported history of mental disorders and a probit regression model of estimate prevalence of CES-D positivity was created as shown in Fig. 1 (Step 2 in Supplementary Fig. 1).

Participants without mental disorders (MD) including depression, bipolar disorder, anxiety disorder, post-traumatic stress disorder, schizophrenia, and dementia (n = 8024) were stratified into risk levels of depression based on sex, age, cohabiting family, social isolation, and bereavement. Sex has been determined to be a risk factor for depression, with a 5.1% prevalence among women, which is higher compared to men (3.6%)^8^. Age is another risk factor, and the higher prevalence in the working generation, especially in middle-aged and older adults, has been noted as compared to retirees^8^. Lifetime depression in middle-aged and older adults has been associated with cohabiting family members^25^. In this study^25^, living with a spouse or partner was associated with significantly lower odds of lifetime depression (OR = 0.67, 95% CI 0.62–0.74). The presence or absence of social isolation was determined using the Lubben Social Network Scale^31^ (LSNS-6) questionnaire, and a score of less than 12 was defined as social isolation. Correlation between CES-D and LSNS-6 has been reported in this cohort^26^.

After stratifying the participants into subgroups of working and retired generations with a cutoff of 65 years age, they were stratified by sex. Of the individuals in these subgroups, 7827 people who answered the “number of cohabiting family members” question were stratified by the factor of cohabiting family members into 8 subgroups, followed by probit regression modeling represented in Fig. 2A (Step 4 in Supplementary Fig. 1). Out of the eight subgroups, 7143 people who answered the questionnaire on LSNS-6 and the question regarding bereavement of a close person were stratified into the subgroups “with or without social isolation” and experience of bereavement into 32 subgroups, followed by modeling as shown in Fig. 2B (Step 5 in Supplementary Fig. 1).

### Statistical model and its visualization

R software was used for all the calculations and graph creation. Statistical models for visualization comprised a type of generalized linear model, a probit regression model, which follows the Bernoulli distribution. Only the link function differed from the logistic regression model commonly used in epidemiology. The objective variable was the binarized CES-D score according to a cutoff of 16 points, 1 for depressive symptoms and 0 otherwise, which was used as a surrogate outcome for depression, while the explanatory variable comprised the AIS score, which is an index of insomnia. In this model, the estimated prevalence of CES-D positivity P (CES-D positive | AIS = x) at specific AIS scores was estimated using CES-D outcomes and hypothetical AIS continuous values by fitting with the glm function of R.

Risk factors other than insomnia were used as categorical variables. The population was stratified into subgroups for each category, followed by constructing models for each subgroup. This procedure enables control of confounding factors used for categorization and suppresses the problematic multicollinearity in multiple logistic regression models. The cutoff value of the CES-D score was 16 points, while that of the LSNS-6 score was 12 points. Using the glm function of R, each single regression model and its coefficient with both p-values and 95% confidence intervals were estimated, which are listed in Supplementary Tables 1 and 2. We defined ACP50 as the AIS score at which estimated prevalence of CES-D was 50% and the ACP75 as the AIS score at which that was 75%. They are listed in Supplementary Tables 1 and 2.

Two models in Supplementary Table 1, model “#0xxxxx” of the subgroup without MD, P (CES-D positive | AIS = x, mental disorder = 0) and model #1xxxxx of the subgroup with MD, P (CES-D positive | AIS = x, mental disorder = 1) are visualized as graphs in Fig. 1. Eight models in Supplementary Table 1, P (CES-D positive | AIS = x, mental disorder = 0, stratified by age, sex, and cohabiting family), were visualized in two graphs divided into the working generation and the retired generations shown in Fig. 2A. In Fig. 2B, 25 of the 32 models in Supplementary Table 2, P (CES-D positive | AIS = x, mental disorder = 0, stratified by age, sex, cohabiting family, social isolation, and bereavement), were visualized. A cutoff value of the LSNS-6 score was used 12 points, an index of social isolation. Among the 32 stratified subgroups, the #010101 subgroup did not include participants who were CES-D positive, whereas #000111 included only four people; thus, these models could not be constructed. These two models and the five models with a coefficient of *p* > 0.05 that did not satisfy the statistical significance were excluded from the analysis but the 2 models for those with and without MD were added. These 27 models are visualized in Fig. 2B as heatmaps.

### Prevalence of subgroups categorized by sleep disorder cutoff

The prevalence of CES-D positivity calculated for 7,021 members categorized into the 27 subgroups is visualized in Fig. 2B (Table 2). First, the prevalence of CES-D positivity among 7,021 people without stratification was calculated; then we calculated the prevalence in two subgroups stratified by the sleep disorder cutoff of 6 points according to the AIS questionnaire (Table 3). Next, these subjects were stratified into eight subgroups by sex, age, and cohabitance with other family members, and each subgroup was divided by the ACP50 cutoff. Finally, the number of CES-D positive cases in each subgroup was estimated. These subgroups were then divided into two categories of below and above ACP50, and the observed prevalence of each category was calculated from the total number of people in each category and the total number of CES-D-positive participants (Table 2). Furthermore, the subjects were stratified into 32 subgroups by sex, age, cohabiting family members, social isolation, and bereavement of close persons, and each subgroup was divided by the ACP50 cutoff. The prevalence of categories of below and above ACP50 was calculated in the same manner (Table 2). Prevalence was also calculated using the ACP75 cutoff (Table 2).

### Effect of stratification on screening

At the AIS score of 6 points, which is considered the sleep disorder category, the estimated prevalence in the population without MD, P (CES-D positive | AIS = 6, mental disorder = 0), was calculated to be 0.283. Using this ACP of 28.3 as a cutoff in each model, we examined the total number of people above or below ACP28.3 and the number of CES-D positive individuals in each stratified group and added them together. From these values, each prevalence was calculated (Table 3). When stratified by three or five risk factors, the similar number of CES-D-positive individuals were calculated with a detectable cutoff ACP in increments of 0.1 to determine the ACP value with the performance closest to that of ACP26 for three stratifications and ACP25 for five stratifications, respectively.

## Supplementary Information

Supplementary Information 1.

Supplementary Information 2.

Supplementary Information 3.

Supplementary Information 4.

Supplementary Information 5.

Supplementary Information 6.

Supplementary Information 7.

## Data Availability

The data that support the findings of this study are available from Tohoku Medical Megabank Organization, but restrictions apply to the availability of these data, which were used under permission for the current study, and so are not publicly available. Data are however available from the authors upon reasonable request and with permission of Tohoku Medical Megabank Organization.
