# Supplementary material for: Visualization of estimated prevalence of CES-D positivity accounting for background factors and AIS scores
Source: Sci Rep. 2022 Oct 21;12:17656. doi: 10.1038/s41598-022-22266-1 (PMC9586984; doi:10.1038/s41598-022-22266-1)
Supplement: Supplementary file 1 — Supplementary Information 1. [file 41598_2022_22266_MOESM1_ESM.docx]

Supplementary Table 1. Representative values of the model shown in Figures 1 and 2A.

| - | Model# | Sample size (n) | ACP50 | ACP75 | Regression Coefficient | | | |
| --- | --- | --- | --- | --- | --- | --- | --- | --- |
|  |  |  |  |  | Estimate | p value | 95% CI | |
|  |  |  |  |  |  |  | 2.5% | 97.5% |
| Figure 1 | #0xxxxx | 8,024 | 8.8 | 12.2 | 0.203 | <0.001 | 0.191 | 0.214 |
|  | #1xxxxx | 416 | 6.3 | 9.3 | 0.228 | <0.001 | 0.187 | 0.271 |
| Figure 2A | #0000xx | 1,391 | 10.0 | 13.2 | 0.207 | <0.001 | 0.172 | 0.244 |
|  | #0001xx | 85 | 7.2 | 9.5 | 0.292 | <0.001 | 0.156 | 0.451 |
|  | #0010xx | 1,837 | 10.0 | 13.7 | 0.185 | <0.001 | 0.163 | 0.208 |
|  | #0011xx | 239 | 9.4 | 13.2 | 0.177 | <0.001 | 0.115 | 0.243 |
|  | #0100xx | 1,107 | 7.9 | 10.7 | 0.242 | <0.001 | 0.207 | 0.278 |
|  | #0101xx | 94 | 9.1 | 12.1 | 0.226 | <0.001 | 0.120 | 0.349 |
|  | #0110xx | 2,924 | 8.3 | 11.7 | 0.200 | <0.001 | 0.181 | 0.218 |
|  | #0111xx | 148 | 6.1 | 9.2 | 0.216 | <0.001 | 0.142 | 0.298 |

Supplementary Table 2. Representative values of the model shown in Figure 2B.

| Model# | Sample size (n) | ACP50 | ACP75 | Regression Coefficient | | | |
| --- | --- | --- | --- | --- | --- | --- | --- |
|  |  |  |  | Estimate | p value | 95% CI | |
|  |  |  |  |  |  | 2.5% | 97.5% |
| #000000 | 535 | 11.1 | 14.6 | 0.194 | <0.001 | 0.125 | 0.266 |
| #000001 | 435 | 9.5 | 12.7 | 0.214 | <0.001 | 0.155 | 0.276 |
| #000010 | 182 | 11.0 | 14.5 | 0.191 | <0.001 | 0.102 | 0.286 |
| #000011 | 90 | 10.2 | 13.9 | 0.181 | 2.26E-03 | 0.066 | 0.307 |
| #000100 | 29 | 7.4 | 10.5 | 0.220 | 4.09E-02 | 0.017 | 0.442 |
| #000101^†^ | 31 | n.c. | n.c. | n.c. | n.c. | n.c. | n.c. |
| #000110^†^ | 14 | 8.9 | 12.1 | 0.209 | 0.1295* | -0.045 | 0.542 |
| #000111^†^ | 4 | n.c. | n.c. | n.c. | n.c. | n.c. | n.c. |
| #001000 | 681 | 10.8 | 14.4 | 0.187 | <0.001 | 0.146 | 0.229 |
| #001001 | 699 | 9.1 | 12.1 | 0.219 | <0.001 | 0.180 | 0.261 |
| #001010 | 175 | 12.5 | 19.3 | 0.099 | <0.001 | 0.043 | 0.156 |
| #001011 | 81 | 7.2 | 10.0 | 0.244 | <0.001 | 0.152 | 0.348 |
| #001100 | 105 | 9.0 | 12.0 | 0.220 | 1.02E-03 | 0.092 | 0.359 |
| #001101 | 64 | 10.6 | 16.2 | 0.121 | 3.20E-02 | 0.013 | 0.231 |
| #001110 | 29 | 8.7 | 13.1 | 0.153 | 4.44E-02 | 0.010 | 0.317 |
| #001111^†^ | 11 | 7.7 | 9.9 | 0.305 | 0.0866* | -0.010 | 0.732 |
| #010000 | 428 | 8.4 | 11.2 | 0.240 | <0.001 | 0.172 | 0.310 |
| #010001 | 308 | 7.8 | 10.4 | 0.260 | <0.001 | 0.190 | 0.334 |
| #010010 | 199 | 8.1 | 11.6 | 0.195 | <0.001 | 0.130 | 0.263 |
| #010011 | 90 | 6.9 | 9.6 | 0.253 | <0.001 | 0.149 | 0.367 |
| #010100^†^ | 27 | 7.6 | 10.0 | 0.285 | 0.06058* | -0.001 | 0.647 |
| #010101^†^ | 15 | n.c. | n.c. | n.c. | n.c. | n.c. | n.c. |
| #010110 | 31 | 10.5 | 14.8 | 0.157 | 3.49E-02 | 0.021 | 0.327 |
| #010111 | 14 | 6.6 | 8.4 | 0.365 | 2.80E-02 | 0.094 | 0.773 |
| #011000 | 1,231 | 8.6 | 11.9 | 0.206 | <0.001 | 0.174 | 0.239 |
| #011001 | 811 | 9.0 | 13.0 | 0.168 | <0.001 | 0.136 | 0.201 |
| #011010 | 461 | 7.6 | 10.7 | 0.220 | <0.001 | 0.174 | 0.269 |
| #011011 | 222 | 6.4 | 9.5 | 0.221 | <0.001 | 0.161 | 0.285 |
| #011100 | 52 | 7.3 | 10.9 | 0.184 | 3.70E-03 | 0.065 | 0.320 |
| #011101 | 38 | 5.8 | 9.0 | 0.206 | 1.74E-02 | 0.052 | 0.396 |
| #011110 | 31 | 5.7 | 8.3 | 0.260 | 4.94E-03 | 0.095 | 0.459 |
| #011111^†^ | 20 | 4.7 | 9.2 | 0.149 | 0.153* | -0.048 | 0.375 |

† Model is not significant. * *p* > 0.05. n.c., the model was not calculated. In the case of #010101, there is no case of CES-D positive prevalent. In the case of #000111 and #010101, the number of subjects was too small to construct significant model.

Supplementary Table 3. Number of people determined by the cutoff of 16 CES-D points at each AIS score.

w/o MD

| AIS score | | 0 | 1 | 2 | 3 | 4 | 5 | 6 | 7 | 8 | 9 |
| --- | --- | --- | --- | --- | --- | --- | --- | --- | --- | --- | --- |
| CES-D | negative | 950 | 1433 | 1165 | 898 | 660 | 526 | 381 | 249 | 151 | 76 |
|  | positive | 28 | 66 | 93 | 139 | 140 | 142 | 168 | 172 | 143 | 102 |
|  | observed prevalence* (%) | 2.9 | 4.4 | 7.4 | 13.4 | 17.5 | 21.3 | 30.6 | 40.9 | 48.6 | 57.3 |
|  | estimated prevalence† (%) | 3.7 | 5.6 | 8.3 | 11.9 | 16.4 | 21.9 | 28.3 | 35.5 | 43.3 | 51.4 |

| 10 | 11 | 12 | 13 | 14 | 15 | 16 | 17 | 18 | 19 | 20 | 21 | 22 | 23 | 24 |
| --- | --- | --- | --- | --- | --- | --- | --- | --- | --- | --- | --- | --- | --- | --- |
| 45 | 34 | 17 | 11 | 5 | 6 | 3 | 3 | 2 | 0 | 0 | 0 | 0 | 0 | 0 |
| 61 | 44 | 24 | 27 | 20 | 16 | 7 | 9 | 4 | 3 | 0 | 1 | 0 | 0 | 0 |
| 57.5 | 56.4 | 58.5 | 71.1 | 80.0 | 72.7 | 70.0 | 75.0 | 66.7 | 100.0 | - | 100.0 | - | - | - |
| 59.4 | 67.0 | 74.0 | 80.1 | 85.3 | 89.4 | 92.7 | 95.1 | 96.8 | 98.0 | 98.8 | 98.9 | 99.3 | 99.6 | 99.9 |

MD

| AIS score | | 0 | 1 | 2 | 3 | 4 | 5 | 6 | 7 | 8 | 9 |
| --- | --- | --- | --- | --- | --- | --- | --- | --- | --- | --- | --- |
| CES-D | negative | 19 | 39 | 37 | 32 | 28 | 20 | 21 | 14 | 8 | 5 |
|  | positive | 4 | 1 | 3 | 7 | 14 | 16 | 15 | 24 | 28 | 15 |
|  | observed prevalence* (%) | 17.4 | 2.5 | 7.5 | 17.9 | 33.3 | 44.4 | 41.7 | 63.2 | 77.8 | 75.0 |
|  | estimated prevalence† (%) | 7.4 | 11.1 | 16.1 | 22.3 | 29.6 | 37.9 | 46.9 | 55.9 | 64.7 | 72.8 |

| 10 | 11 | 12 | 13 | 14 | 15 | 16 | 17 | 18 | 19 | 20 | 21 | 22 | 23 | 24 |
| --- | --- | --- | --- | --- | --- | --- | --- | --- | --- | --- | --- | --- | --- | --- |
| 2 | 1 | 2 | 1 | 2 | 1 | 0 | 0 | 0 | 0 | 0 | 0 | 0 | 0 | 0 |
| 15 | 11 | 6 | 7 | 5 | 8 | 2 | 1 | 0 | 1 | 1 | 0 | 0 | 0 | 0 |
| 88.2 | 91.7 | 75.0 | 87.5 | 71.4 | 88.9 | 100.0 | 100.0 | - | 100.0 | 100.0 | - | - | - | - |
| 79.8 | 85.6 | 90.2 | 93.6 | 96.0 | 97.6 | 98.6 | 99.2 | 99.6 | 99.8 | 100.0 | 100.0 | 100.0 | 100.0 | 100.0 |

*Observed Prevalence was calculated at each AIS score.

†Each estimated prevalence represents P (CES-D positive | AIS = x) calculated from the model.
