# Supplementary material for: Visualization of estimated prevalence of CES-D positivity accounting for background factors and AIS scores
Source: Sci Rep. 2022 Oct 21;12:17656. doi: 10.1038/s41598-022-22266-1 (PMC9586984; doi:10.1038/s41598-022-22266-1)
Supplement: Supplementary file 7 — Supplementary Information 7. [file 41598_2022_22266_MOESM7_ESM.pdf]

9,815 participants ≥ 20 years old

↓ Exclude missing any answers from AIS and CES-D (1315)

Step1:
Crude (8,440)

Crude	8,440

Step2:
Stratification by History of Mental Disorder (MD) (8,440) used in Fig.1 and Table 1

w/o	8,024	#0xxxxx
With	416	#1xxxxx

↓ Exclude participants with history of MD (416)

Step3:
Stratification by age and sex (8,024)

	Working generation <65 years (Working)	Retired generation > 64 years (Retired)
Male	1,235	1,524
Female	3,132	2,133

↓ Exclude missing answers from the number of cohabiting family (199)

Step4:
Stratification by age, sex, and cohabiting family (7,825) used in Fig.2A

	Working Male		Retired Male		Working Female		Retired Female	
Cohabiting	1,107	#0100xx	1,391	#0000xx	2,924	#0110xx	1,837	#0010xx
Alone	94	#0101xx	85	#0001xx	148	#0111xx	239	#0011xx

↓ Exclude missing any answers from LSNS-6 (346) and bereavement of closed persons (336)

Step5:
Stratification by age, sex, cohabiting family, social isolation, and bereavement (7,143) used in Fig.2B and Table 2

Participants with Social Isolation Negative LSNS-6 score 12≥

Bereavement	Working, Male, Cohabiting		Working, Male, Alone		Retired, Male, Cohabiting		Retired, Male, Alone	
Negative	428	#010000	27	#010100	535	#000000	29	#000100
Positive	308	#010001	15	#010101	435	#000001	31	#000101

Bereavement	Working, Female, Cohabiting		Working, Female, Alone		Retired, Female, Cohabiting		Retired, Female, Alone	
Negative	1,231	#011000	52	#011100	681	#001000	105	#001100
Positive	811	#011001	38	#011101	699	#001001	64	#001101

Participants with Social Isolation Positive LSNS-6 score <12

Bereavement	Working, Male, Cohabiting		Working, Male, Alone		Retired, Male, Cohabiting		Retired, Male, Alone	
Negative	199	#010010	31	#010110	182	#000010	14	#000110
Positive	90	#010011	14	#010111	90	#000011	4	#000111

Bereavement	Working, Female, Cohabiting		Working, Female, Alone		Retired, Female, Cohabiting		Retired, Female, Alone	
Negative	461	#011010	31	#011110	175	#001010	29	#001110
Positive	222	#011011	20	#011111	81	#001011	11	#001111

Figure S1. Scheme of stratification of participants
